# Potentially pathogenic *Acanthamoeba* genotype T4 isolated from dental units and emergency combination showers

**DOI:** 10.1590/0074-02760170147

**Published:** 2017-12

**Authors:** Esteban Castro-Artavia, Lissette Retana-Moreira, Jacob Lorenzo-Morales, Elizabeth Abrahams-Sandí

**Affiliations:** 1 University of Costa Rica, Faculty of Microbiology, Department of Parasitology, San Pedro, San José, Costa Rica; 2 University of Costa Rica, Centro de Investigación en Enfermedades Tropicales, San Pedro, San José, Costa Rica; 3 University of La Laguna, Institute of Tropical Diseases and Public Health of the Canary Islands, Tenerife, Spain

**Keywords:** *Acanthamoeba* genotype T4, free-living amoeba, dental unit, emergency combination showers

## Abstract

**BACKGROUND:**

*Acanthamoeba* is the genus of free-living amoebae that is most frequently isolated in nature. To date, 20 *Acanthamoeba* genotypes have been described. Genotype T4 is responsible for approximately 90% of encephalitis and keratitis cases. Due to the ubiquitous presence of amoebae, isolation from environmental sources is not uncommon; to determine the clinical importance of an isolation, it is necessary to have evidence of the pathogenic potential of amoebae.

**OBJECTIVE:**

The aim of this study was to physiologically characterise 8 *Acanthamoeba* T4 isolates obtained from dental units and emergency combination showers and to determine their pathogenic potential by employing different laboratory techniques.

**METHODS:**

Eight axenic cultures of *Acanthamoeba* genotype T4 were used in pathogenic potential assays. Osmotolerance, thermotolerance, determination and characterisation of extracellular proteases and evaluation of cytopathic effects in MDCK cells were performed.

**FINDINGS:**

All of the isolates were osmotolerant, thermotolerant and had serine proteases from 44-122 kDa. Two isolates had cytopathic effects on the MDCK cell monolayer.

**MAIN CONCLUSION:**

The presence of *Acanthamoeba* T4 with pathogenic potential in areas such as those tested in this study reaffirms the need for adequate cleaning and maintenance protocols to reduce the possibility of infection with free-living amoebae.

*Acanthamoeba* is a free-living amoebae (FLA) of worldwide distribution, commonly found in soil and water sources. Based on rRNA sequences, this genus is divided into 20 different genotypes (Corsaro et al. 2015), known as T1 to T20. Genotype T4 is the most frequently isolated genotype from clinical cases (Khan 2006, Maciver et al. 2013). It has been reported that more than 90% of amoebic keratitis cases have been linked with this genotype, with similar percentages for granulomatous amoebic encephalitis and cutaneous infections (Khan 2006). Other isolated genotypes linked to clinical manifestations include T2, T3, T5, T6, T11 and T15 (Di Cave et al. 2008, Walochnik et al. 2008, Ledee et al. 2009, Lorenzo-Morales et al. 2011, Omaña-Molina et al. 2016). In addition to their capacity to produce damage, some *Acanthamoeba* trophozoites harbour pathogenic bacteria, which are resistant to the lytic mechanisms of the amoeba. Reports include *Legionella* sp, *Franciscella tularensis,* nontuberculous mycobacteria, and others (Greub & Raoult 2004). In fact, it has been postulated that amoebae could be considered biological incubators or environmental reservoirs of these bacteria, acting as “Trojan horses” and increasing the transmission potential for humans (Janda 2010).

Due to the ubiquitous presence of amoebae, isolation from environmental sources is common. To date, several studies report *Acanthamoeba* in soil (especially genotype T4), swimming pools, water networks from hospitals, and air conditioning units (Thomas et al. 2006, Chan et al. 2011, Fabres et al. 2016, Reyes-Batlle et al. 2016), as well as equipment and devices for medical treatments or emergency situations. This equipment includes dialysis and dental units (Dendana et al. 2008, Trabelsi et al. 2010, Retana-Moreira et al. 2015), as well as combination shower units (Retana-Moreira et al. 2014).

Different laboratory techniques have been employed to demonstrate the presence of characteristics considered necessary for the amoebae to be potentially pathogenic (Khan 2006). Some of the techniques include thermotolerance and osmotolerance assays, as well as the secretion of extracellular proteases and the evaluation of cytopathic effects in cell culture. In this study, we employed these techniques in eight axenic isolates of *Acanthamoeba* T4 from dental and emergency combination showers to determine their pathogenic potential.

## MATERIALS AND METHODS

*Samples -* A total of eight axenic isolates of *Acanthamoeba* genotype T4 were employed in this study. Samples were obtained from dental units (DU3, DU4, DU5, DU6, DU8, DU12) (Retana-Moreira et al. 2015) and emergency combination showers (CSU7 and CSUT7) (Retana-Moreira et al. 2014). Each axenic isolate was grown on 25 cm^2^ tissue culture flasks (Cellstar, Greiner Bio-One GmbH, Frickenhausen, Germany) with 5 mL of PYG medium (0.75% proteose peptone, 0.75% yeast extract and 1.5% glucose) containing 10 μg/mL gentamicin (Sigma Aldrich Co., St. Louis, USA). Isolates were maintained under aerobic conditions at 30°C without shaking.

*Acanthamoeba* Neff (ATCC30010) and the isolate CLC-16 genotype T3 (Martín-Navarro et al. 2010) were used as positive controls.

*Osmotolerance and thermotolerance assays* The amoebae were cultivated on non-nutrient agar plates with 0.5 M and 1 M mannitol, supplemented with *Escherichia coli.* Approximately 1000 trophozoites were separately inoculated at the centre of the agar plate and incubated at 30°C for up to 72 h with daily observation. Proliferation of *Acanthamoeba* sp. was observed by measuring the increase in diameter of the clearance zone in the bacterial lawn.

For thermotolerance assays, the same quantity of amoebae was inoculated onto non-nutrient agar plates supplemented with *E. coli.* Cultures were incubated at 37°C and 40°C. Proliferation of *Acanthamoeba* sp. was evaluated by inverted microscopic examination daily for up to 72 h. Each assay was performed in triplicate.

*Preparation of Acanthamoeba conditioned medium (ACM) -* ACM was prepared by incubating amoebae confluent flasks with 5 mL of RPMI-1640 medium (Sigma Aldrich Co., St. Louis, USA) at 30°C for 24 h. After this time, cell-free conditioned medium was collected by centrifugation at 15000 rpm for 10 min, filtered through a 0.22 μm pore filter (Minisart, Sartorius stedim Biotech GmbH, Goettingen, Germany) and stored at −80°C until use.

*Determination and characterisation of protease secretion by zymography -* Zymographic assays were performed as previously described by Herron et al. (1986). Briefly, ACM was mixed with the sample buffer (10% sodium dodecyl sulphate, 4% sucrose, 0.25 mM Tris-HCl and 0.1% bromofenol, pH 6.8) and underwent electrophoresis on SDS-polyacrylamide gels (SDS-PAGE) containing gelatin (1 mg/mL). After electrophoresis, gels were washed twice in 2.5% Triton X-100 (w/v) for 30 min to remove the SDS. Finally, gels were incubated overnight, at 37°C, in a developing buffer (50 mM Tris-HCl, pH 8.0, containing 10 mM CaCl_2_), and rinsed and stained with Coomassie brilliant blue R-250 (Bio-Rad Laboratories, California, USA). Areas of gelatin digestion, which indicate protease activity, were seen as non-staining regions in the gel.

To characterise the type of protease produced, samples were pre-treated for 30 minutes with phenylmethylsulfonyl fluoride (PMSF, 1 mM final concentration) or 1, 10-phenanthroline (10 mM final concentration) (Sigma Aldrich Co., St. Louis, USA) before electrophoresis. As 1,10-phenanthroline is a reversible inhibitor, it was also included in the developing buffer.

*Evaluation of the in vitro effect of Acanthamoeba on cell culture -* Cell culture: Madin-Darby canine kidney (MDCK) epithelial cells (NBL2 ATCC CCL-34TM) were grown in 75 cm^2^ cell culture flasks (Corning, Corning Incorporated, NY, USA) containing RPMI 1640 medium (Sigma-Aldrich Co., ST. Louis, USA) with penicillin (100 U/mL), streptomycin (100 pg/mL) and 10% foetal calf serum (Gibco, GranIsland, NY, USA). Flasks were maintained at 37°C in a humidified 5% CO_2_ incubator.

Crystal violet stain: MDCK cells were grown until confluence on 24-well plates. Then, *Acanthamoeba* isolates (5 × 10^5^ amoebae per well) were incubated with cell monolayers in serum-free media for 24 h at 37°C. The cytopathic effect was assessed macroscopically after crystal violet staining of the wells. The strains *Acanthamoeba castellanii* Neff American Type Culture Collection 30010) and *Acanthamoeba* T3 strain CLC-16 (Martín-Navarro et al. 2010) were employed as positive controls.

Cytotoxicity assay: MDCK cells were incubated with each *Acanthamoeba* isolate as described above. At the end of the incubation period, supernatants were collected. Cytotoxicity was determined by measuring lactate dehydrogenase (LDH) release at 590 nm, following the indications of the commercial kit manufacturers (LDH BR, Linear Chemicals, Barcelona, Spain).

The percentage of LDH released (% cytotoxicity) was calculated as follows: [LDH activity in experimental sample (measured by optical density at 590 nm) - LDH activity in control samples]/(total LDH activity release - LDH activity in control samples) × 100. Control sample values were obtained from host cells incubated in RPMI 1640. Total LDH release was determined from host cells treated with 1% Triton X-100 for 30 min at 37°C.

Strains *A. castellanii* Neff (ATCC) 30010 and *Acanthamoeba* T3 strain CLC-16 (Martín-Navarro et al. 2010) were employed as positive controls.

## RESULTS

The eight axenic isolates employed in this study were able to grow in plates with 0.5 M and 1.0 M mannitol. The thermotolerance assay showed the same result, as 100% of the isolates grew when incubated at temperatures of 37°C and 40°C.

**Fig. 1:**
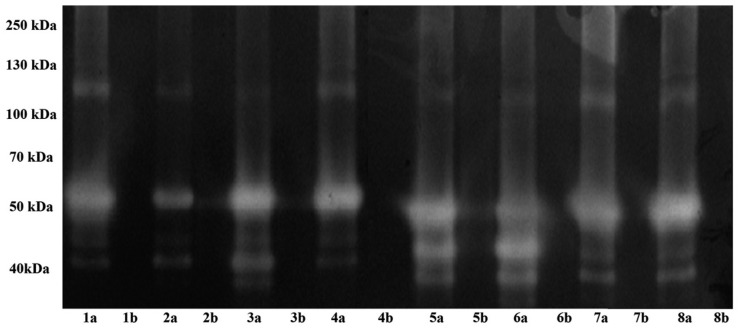
protease zymograms of the eight environmental *Acanthamoeba* isolates. Lanes (a) *Acanthamoeba* conditioned medium (ACM); lanes (b) ACM incubated with serine protease inhibitor [phenylmethylsulfonyl fluoride (PMSF)]. 1: DU12; 2: DU5; 3: DU3; 4: DU8; 5: CSUT7; 6: CSU7; 7: DU6; 8: DU4.

Regarding the secretion of extracellular proteases, ACM from all *Acanthamoeba* isolates showed similar banding patterns on gelatin gels (Fig. 1). Four bands, with molecular weights of approximately 44, 46, 58 and 124 kDa, were observed. All proteases were inhibited when incubating with PMSF 1 mM. In contrast, none of the bands were inhibited by 1, 10-phenanthroline 10 mM. The results suggest the secretion of serine proteases in all isolates.

**Fig. 2:**
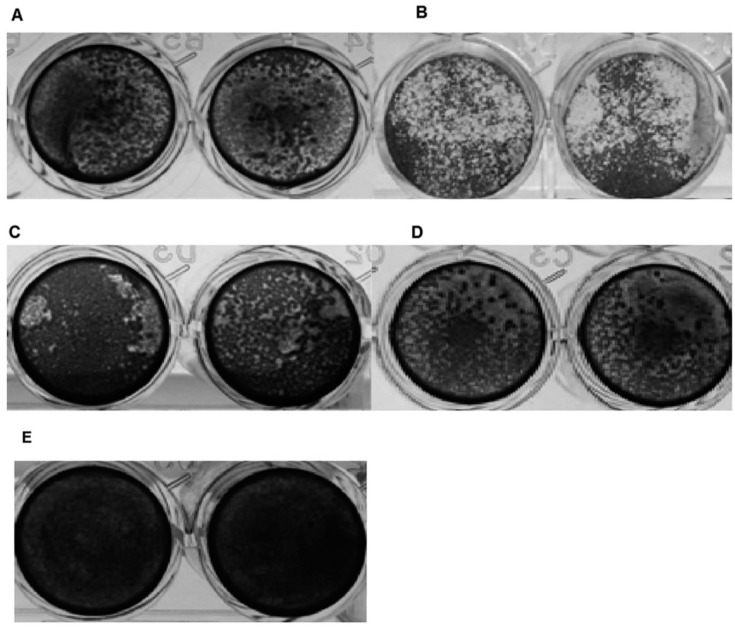
crystal violet staining showed the cytopathic effect of three *Acanthamoeba* environmental isolates on Madin-Darby canine kidney (MDCK) cells. Amoebae were incubated in 24-multiwell plates containing MDCK for 24 h at 37°C. (A) CSUT7; (B) control strain *Acanthamoeba* Neff; (C) DU12; (D) control strain *Acanthamoeba* CLC-16; (E) cell control. Photos are representative of triplicate experiments.

An apparent damage of the cell monolayer was observed only after incubation of the cells with isolates CSUT7 and DU12. The remaining isolates did not have significant monolayer alterations. Fig. 2 shows the crystal violet staining of cells incubated with the *Acanthamoeba* isolates mentioned above, as well as the results of the control strains.

In the LDH release assay, six isolates showed a percentage ≤ 10%. For isolates CSUT7 and DU12, the obtained percentages were 24.4% and 14.4%, respectively. Control strains *Acanthamoeba* Neff and CLC-16 showed percentages of cytotoxicity of 60.9% and 38.8%, respectively.

## DISCUSSION

*Acanthamoeba* is the genus of FLA most frequently found in nature; nevertheless, not every species or genotype is able to produce damage to a host (Schuster & Visvesvara 2004). In previous studies performed in our laboratory, we isolated and axenically grew eight *Acanthamoeba* genotype T4 from dental units and combined emergency units (Retana-Moreira et al. 2014, 2015). Considering the location from which they were isolated and, since this genotype is the most frequently associated with clinical cases (Khan 2006), we considered it relevant to physiologically and biologically characterise the isolates through a series of laboratory assays to determine characteristics associated with pathogenic potential.

The results showed that all *Acanthamoeba* genotype T4 employed in this study were osmotolerant and thermotolerant, which are indirect factors related to pathogenicity (Khan 2001). Growth at high mannitol concentrations has been associated with the ability to resist high osmotic pressures, a situation that the amoebae could face when they act as parasites of the corneal epithelium (Siddiqui & Khan 2012). Thermotolerance is related to the ability of the amoeba to resist normal body temperature or even fever episodes in the host. Moreover, the growth of amoebae at temperatures above 40°C is directly correlated to their capacity to produce cellular damage *in vitro* (Griffin 1972, Walochnik et al. 2000). Even if these two characteristics give the amoeba adaptative advantages when parasitising a host, the mere presence or absence of these characteristics is not enough to define the amoeba as pathogenic. For instance, different species of *Acanthamoeba* may be thermotolerant but non-pathogenic (Schuster & Visvesvara 2004). This characteristic is also less indicative of pathogenicity for amoebae that infect the human cornea because the corneal temperature is approximately 32-35°C; therefore, colonisation by the amoeba could be possible even when the organism is not able to grow at temperatures above 37°C (Tolero et al. 2011).

In this study, we demonstrated the production of extracellular serine proteases by all of the environmental isolates. Migration patterns showed bands with molecular weights from 44 kDa to 124 kDa. These results are similar to the ones obtained for other *Acanthamoeba,* including isolates from clinical cases (Cao et al. 1998, Alfieri et al. 2000). Early reports considered serine proteases as pathogenicity markers (Khan et al. 2000), with proven action of proteases in plasminogen activation, as well as collagen and fibronectin degradation (Khan 2006). Recently, the role of proteases in tissue invasion has been confirmed, emphasising their participation in extracellular matrix digestion but not in the direct process of cellular lysis (Omaña-Molina et al. 2013). It is important to highlight that the activity of these enzymes has been demonstrated particularly in *Acanthamoeba* isolated from clinical cases of encephalitis or keratitis. For environmental isolates (such as the ones employed in this study), a role of proteases in nutrition and encystment/excystment has been proposed (Dudley et al. 2008), considering their presence as necessary for eventual facultative parasitism by the amoeba.

To evaluate the possible *in vitro* cytotoxicity and cytopathic effect of our isolates, we used MDCK cells, a model of cytolytic activity frequently employed in this type of study. By crystal violet staining, it was possible to observe damage to the monolayer 24 h after adding DU12 and CSUT7 isolates or the control strains, *A. castellani* Neff and *Acanthamaoeba* CLC-16. The main observed effect was the disaggregation of the monolayer by amoebae trophozoites that were attached to the plate in the spaces previously occupied by cells or between the attached cells. When using the LDH release assay, these same isolates and control strains showed a level of cytotoxicity that correlated with the degree of cellular damage observed in the crystal violet assay. In a previous study employing different cell lines, Martín-Navarro et al. (2010) reported percentages of cytotoxicity for *Acanthamoeba* Neff of 60% to 70% and for CLC-16, 55% to 75%. These percentages, especially for CLC-16, were significantly higher than those obtained in our study, which could be directly related to the use of the MDCK line. Despite the above mentioned study, and considering that *Acanthamoeba* Neff has a degree of cytotoxicity which could be categorised as moderate to high, our results suggest that the environmental isolates DU12 and CSUT7 showed an *in vitro* cytopathic effect that, although mild, reveals the capability of the amoebae to damage the cell monolayer.

Our study demonstrated virulence factors that suggest a pathogenic potential for all of the *Acanthamoeba* genotype T4 environmental isolates: osmotolerance, thermotolerance and secretion of extracellular proteases. Additionally, two of them (one from a dental unit and one from an emergency combined shower) showed *in vitro* cytopathic effects. The isolation of these types of amoebae in equipment and biosafety devices such as those tested in this study reaffirms the need for adequate cleaning and maintenance protocols to reduce the possibility of infection with FLA.
